# Longitudinal surveillance of *Aedes aegypti* (Diptera: Culicidae) in urban coastal Kenya: population dynamics, blood feeding frequency and dengue virus infection rates

**DOI:** 10.1038/s41598-025-05408-z

**Published:** 2025-07-01

**Authors:** Josephine Osalla, Louis-Clement Gouagna, Gilbert Rotich, Maureen Nzilani, Penina Safari, Kennedy Senagi, Francis Mutuku, Baldwyn Torto, David P. Tchouassi

**Affiliations:** 1 International Centre of Insect Physiology and Ecology, P.O Box 30772-00100, Nairobi, Kenya; 2 MIVEGEC, Université de Montpellier, IRD, CNRS, Montpellier, France; 3 Technical University of Mombasa, Mombasa, Kenya

**Keywords:** *Aedes aegypti*, Dengue-2 virus, Urbanization, Feeding patterns, Climate change, Arbovirus surveillance, Ecology, Microbiology, Climate sciences

## Abstract

The coastal region of Kenya has emerged as a focal point for urban dengue virus transmission driven by *Aedes aegypti* as the primary vector. To gain a deeper understanding of the epidemiological situation, we carried out a year-long longitudinal study (December 2021- November 2022) of the population dynamics of *A. aegypti* through weekly mosquito surveys using ovitrap and CO_2_-baited Biogents (BG) mosquito traps in Ukunda, an urban township in the coastal region. *Aedes* eggs laid in ovitraps were exclusively *A. aegypti* with 80.8% mean hatch rate. A total of 35,109 adult *A. aegypti* were captured, with twice as many females than males. The density of adult *A. aegypti* trap captures varied monthly, but there was no discernible delineation by season. *Aedes aegypti* fed more on humans (human blood index = 0.72). Two dengue-2 virus RNA was detected in two blood-fed specimens that had fed on humans. Multiple linear regression model indicated 59% variation in adult female abundance explained by weather variables including daily range in wind speed (82.9%) and temperature (17.1%). In contrast, random forest model revealed 83% variation in egg abundance attributed to weather variables, being positively influenced by mean daily temperature, wind speed, relative humidity and negatively by total precipitation. Our results confirm year-round vector presence and active circulation of dengue virus indicative of endemicity in urban Kenya. The findings highlight the importance of short-term climatic factors as predictors of *A. aegypti* dynamics of value in surveillance and control of arboviral diseases such as dengue.

**Supplementary Information:**

The online version contains supplementary material available at 10.1038/s41598-025-05408-z.

## Introduction

Dengue is an arboviral disease of public health significance and accounts for an estimated 400 million human cases each year, globally^1^. The disease disproportionately affects people in tropical and sub-tropical regions mostly in urban and semi-urban areas where the primary vector *Aedes aegypti* thrives^2^. Re-emergence of the arboviral diseases in parts of Africa, such as Kenya has been accompanied by frequent outbreaks with notable contribution to febrile ailments impacting on primary health care^3^. Human population growth and climate change are creating conditions for geographic expansion of mosquito vectors and the causative dengue virus. The centrality of *Aedes* mosquitoes’ ecology to the epidemiology of the arboviruses, (e.g. dengue, chikungunya, zika) indicates the likelihood of high sensitivity to environmental variables, including abiotic (e.g., temperature, rainfall, humidity) and other biotic factors. Indeed, precipitation and temperature affect mosquito breeding, and rate of immature development, respectively^2^ and thought to invariably influence virus transmission rates, and arbovirus disease outbreaks including dengue.

Surveillance of potential mosquito species involved in pathogen transmission is a cornerstone of dengue risk assessment and for guiding interventions. Vector surveillance is undertaken on the assumption that a positive correlation exists between vector ecology, their population density and incidence of human cases^4^. However, this simplified relationship may not always hold^5,6^. Indeed, heterogeneity in adult abundance, survival and blood-feeding patterns^7–10^ have been described indicating variations in competence of different populations to virus transmission^11–13^. Surprisingly, despite evidence of human infection in Africa^3,14^, data on dengue infection in vectors are scarce or remain insufficiently and unevenly explored^15,16^. Testing associations between mosquito-derived variables and identifying which specific combinations determinants relate epidemiologically to dengue transmission risk is an important knowledge gap^17^.

The coastal region of Kenya has emerged as a focal point for urban dengue virus transmission, with a history of repeated outbreaks^3^, necessitating a deeper understanding of the factors underlying the epidemiological significance in the region. Despite poor diagnosis, seasonal fluctuations in human infection and outbreak occurrence have been observed^3,18^, with a knowledge gap of the underlying drivers of this trend. In particular, there is only scarce data over longitudinal scale on the *A. aegypti* population dynamics its ecological relation to disease transmission risk and their possible association with climatic factors^5^. Our objective in the current study was to describe the dynamics of aspects of the ecology of *A. aegypti* over one year in Ukunda, an urban township in the South Coast of Kenya endemic for dengue. Specifically, we examined seasonal patterns in adult *A. aegypti* abundance, egg-laying activity, blood-feeding behaviour and dengue virus infection rates and determined the relationship between population attributes (oviposition activity and adult abundance) and climatic changes in the study location. The study advances our understanding of the environmental conditions driving the dynamics of *A. aegypti* population while also providing much useful information about the disease transmission risk within the coastal setting in Kenya.

## Materials and methods

### Ethical considerations

The study was approved by the Scientific Ethics Review Unit of the Kenya Medical Research Institute (SERU No.3312) and all methods were performed in accordance with the relevant guidelines and regulations. Further informed consent (verbal and written) was sought from household heads to set up BG sentinel traps and ovitraps around their homesteads. This entailed home visits, an explanation of the study objectives, and seeking their consent to access their private property for mosquito sampling.

### Study site

**Fig. 1:**
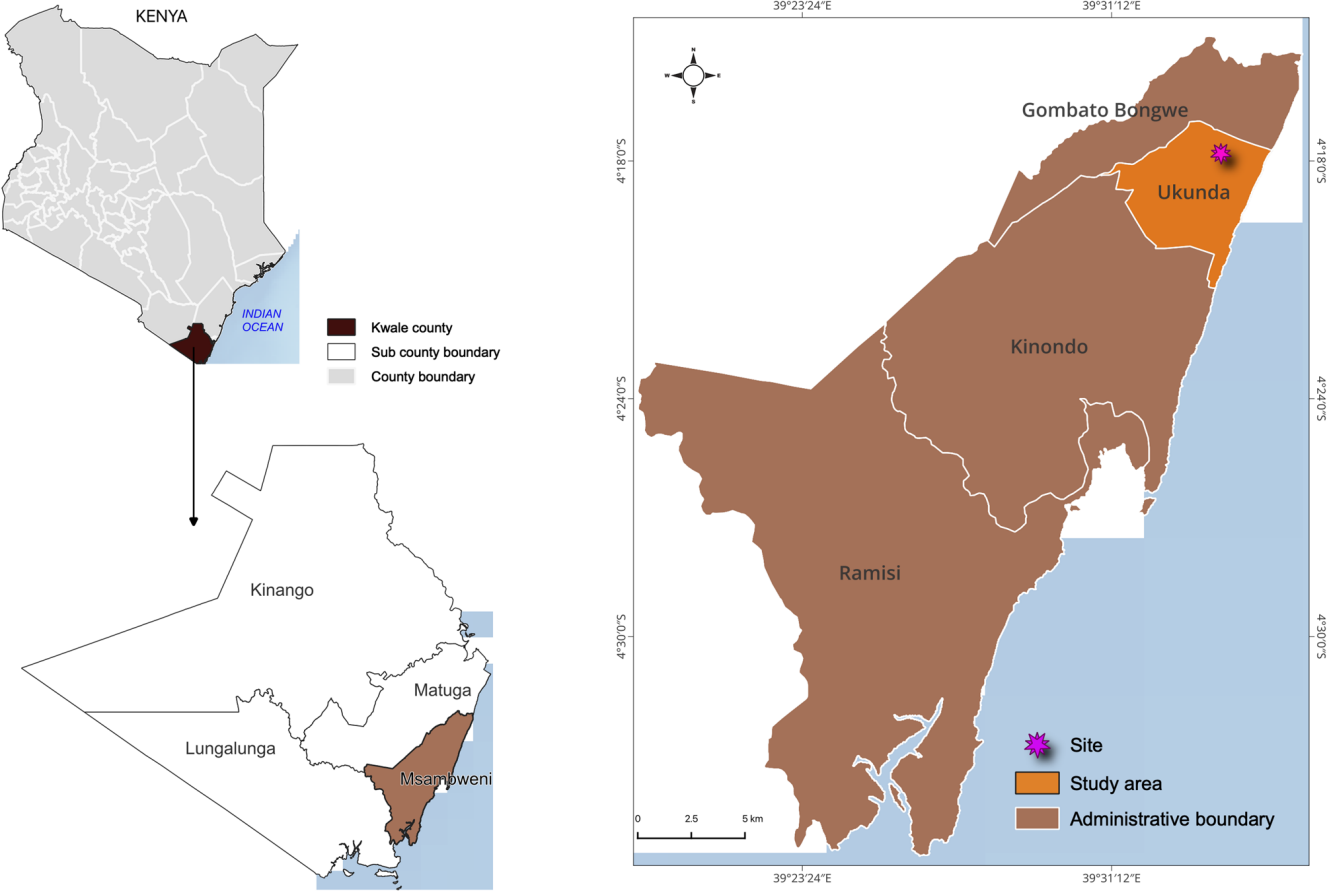
Location of the study site in Kenya.

The study was undertaken in Ukunda (4°16′ 38.8992″ S, 39°34′ 9.0012″ E, altitude 23 m.a.s.l.), an urban town centre located ~ 30 km south of Mombasa in Kwale County along the Indian Ocean coastline of Kenya (Fig. 1). Dengue activity in the area includes its association with febrile illness in children^3^ and abundance of the primary vector *A.* *aegypti*^9^. The tropical climate of the area is characterised by bimodal rainfall with short rains lasting August–October and long rains between March and June.

#### Adult sampling

BG sentinel traps (Biogents AG, Regensburg, Germany) were deployed daily for two to three consecutive days each week to capture adult host-seeking *Aedes* (*Stegomyia*) mosquitoes from December 2021 to November 2022. Each trap was baited with carbon dioxide (CO_2_) generated from a yeast-sugar mixture^19^ and replaced after 24 h. Traps were set at 06:30 h and retrieved at 18:00 h to sample day-time active *Aedes* spp. and/or left 12-h overnight until the following morning at 06: 30 h to target night-time biting mosquitoes. Given previous reports of higher outdoor than indoor activity of this mosquito species in this urban coastal setting^8,9^, traps were placed outdoors around selected homesteads. Sampling was designed to cover a large spatial area (~ 3 × 3 km^2^), moving the traps every second day to a new locality at least 500 m away. Except in December 2021 when only 6 traps were deployed, an average of 12 BG traps were operated each week, amounting to a total of 24 to 60 sampling records each month. After collection and change of collection bag, the collected samples were immobilised using triethylamine, preserved in liquid nitrogen in the field and transported to the laboratory at *icipe*, Nairobi. *Aedes aegypti* among the specimens were identified morphologically using taxonomic keys^20,21^ and then stored at − 80 °C until further processing. The samples were identified on ice packs to preserve the virus for isolation by cell culture.

#### Monitoring oviposition activity

Concurrently, to monitor egg-laying activity by gravid *Aedes* mosquitoes, between 13 and 29 ovitraps were deployed monthly averaging 4/week, except for December 2021 when only 4 ovitraps were deployed. These were set outside selected premises and business areas like restaurants. Each ovitrap consisted of a 1.2-L black plastic pot filled with 650 ml of tap water. The oviposition substrate (ovistrip) was made of a brown germination paper (400HPT Sedburo Equipment, Des Plaines, USA) (measuring 15 × 10 cm) which was placed inside the ovitrap. The papers were labelled on the back with a unique number, followed by the number of the week of collection. Each week, the eggs laid were collected for four consecutive days. The number of eggs from each weekly samples was recorded, based on microscopic observation of number of eggs observed on each ovitrap substrate. Subsequently, egg papers were separately soaked in tap water and hatched larvae were reared to adulthood in a field lab at Msambweni (~ 23 km from Ukunda). Hatched adults were identified to species and scored by sex and then preserved for onward transportation to *icipe* and processing as described above.

### Virus screening in mosquitoes

Field-collected mosquito samples were separated into two lots of unfed specimens and fed based on visual examination of the presence and absence of blood in their abdomens.

The unfed cohorts were pooled (≤ 25 mosquitoes per pool) according to sex, species, and collection date. Individual pools were homogenised in medium (Minimum Essential Medium (MEM), supplemented with 15% fetal bovine serum + 2% L-Glutamine + 2% antibiotic/ antimycotic). The homogenates were clarified by centrifugation at 12,000 rpm (Eppendorf centrifuge 5417R) for 10 min at 4 °C and the supernatants used for virus screening via cell culture and RT-PCR. The homogenate from each pool (140 µl) was subjected to RNA extraction using QIAamp Viral RNA Kit (Catalogue # 52,906) according to the manufacturer’s protocol. In addition, RNA was extracted from individual engorged specimens, while the pellet was subjected to DNA extraction for blood meal analysis. Extracted RNA was reverse transcribed to cDNA using Super Script IV reverse transcriptase (Invitrogen, Carlsbad, CA) and random hexamers followed by RT-PCR using Mytaq PCR Mix (Catalogue # BIO21106). The cDNA was tested using a panel of generic primers (*Alphavirus* and *Flavivirus*) as described^22^. Controls included cDNA of West Nile virus and a no-template in all PCR runs. Amplicons were resolved in 1.5% agarose gel stained with ethidium bromide.

For culture, each pool supernatant (50 μl) from unfed mosquito samples was inoculated in a single well of a 24-well culture plate containing a confluent monolayer of Vero cells (CCL81) grown in minimum essential medium (MEM), which was supplemented with 10% FBS (fetal bovine serum) and 2% L-glutamine and 2% antibiotic/antimycotic solution. The inoculated cultures were incubated for 45 min to allow for virus adsorption, and each sample was maintained in MEM supplemented with 2% FBS and 2% antibiotic/antimycotic solution. The cultures were incubated at 37 °C in 5% CO_2_ and monitored daily, for up to 14 days, for cytopathic effects (CPE) as an indication of virus infection. Supernatants from pools found to be positive by RT-PCR were similarly cultured (in Vero cells and C6/36 cell lines) and monitored for CPE.

### Blood meal analysis

DNA was extracted (ISOLATE II Genomic DNA Kit, Bioline, Meridian Bioscience, Germany) from the pellet of each blood-fed specimen (supernatant used for virus screening) and used to identify the host species from which *A. aegypti* took blood meals by high-resolution melting-PCR using a combination of gene targets in separate PCR runs viz: cytochrome B (cyt b) and 16S rRNA or 12S mitochondrial rRNA gene as described previously^10,23^. However, few samples that could not be reliably scored (due to absence of reliable controls), were amplified for the 12S mitochondrial rRNA gene and then sequenced as well as BLAST query in GenBank as described^10^.

### Weather data

Weather variables during the sampling period were obtained from NASA Power, an open source for weather variables. https://power.larc.nasa.gov/data-access-viewer/. The weather variables considered during the sampling period in Ukunda, coastal Kenya include daily average temperature (in Celsius), diurnal temperature range (DTR) (in Celsius, measured as the difference between max and min temperature in a day), relative humidity (in Percentage), precipitation (in mm), wind speed (in cm/s), and diurnal wind range (DWR) (in cm/s, measured as the difference between max and min wind speed in a day).

### Statistical analysis

Total number of male and female *A. aegypti* was determined for each trap (i.e. density) throughout the study duration. Likewise, the number of *Aedes* eggs found in each ovitraps and the number of *A. aegypti* reared as adults from each ovitrap sample were counted. Variation in the number of adult *A. aegypti* per trap recorded were analysed via Generalized Linear Model fitting a negative binomial distribution with month as the explanatory variable and analysed separately for each sex with mean separation done via emmeans with Tukey method for p-value adjustment. The method of analysis is justified as vector sampling targeted different locations each session (no repeated measures). The same procedure was applied to variations in the number of eggs laid each month. Pearson’s correlation analysis was performed to test for association between captures of adult *A. aegypti* female and male and between *A. aegypti* hatched from collected *Aedes* eggs. Pair-wise comparison in the monthly egg-laying rate was tested by Dunn test with p-values adjusted with the Holm method. The human blood index was calculated as a proportion feeding on humans of the total fed mosquitoes examined.

We analysed the effect of weather variables on the monthly mean abundance of *A. aegypti* female/trap or number of eggs/ovitrap by fitting a multiple linear regression model and then stepwise regression analysis to select and identify the most important variables. The explanatory variables included mean values of daily wind range (DWR), relative humidity, wind speed, temperatures, daily temperature range (DTR), total precipitation and modelled as log transformations (adult abundance data only) to stabilize the variance and linearize the relationships with the response variable. The analyses were done in R v. 4.3.3 and Python. A random forest model (best fit based on mean squared error (MSE) and R^2^ performance metrics) was fitted to explore abundance of eggs laid and the weather variables’ mean values of temperature, relative humidity, wind speed, and total precipitation as independent variables. A time series plot to visualise monthly variation in *A. aegypti* female abundance or number of eggs laid against the individual weather variables was performed.

## Results

### Mosquito composition and abundance

**Fig. 2:**
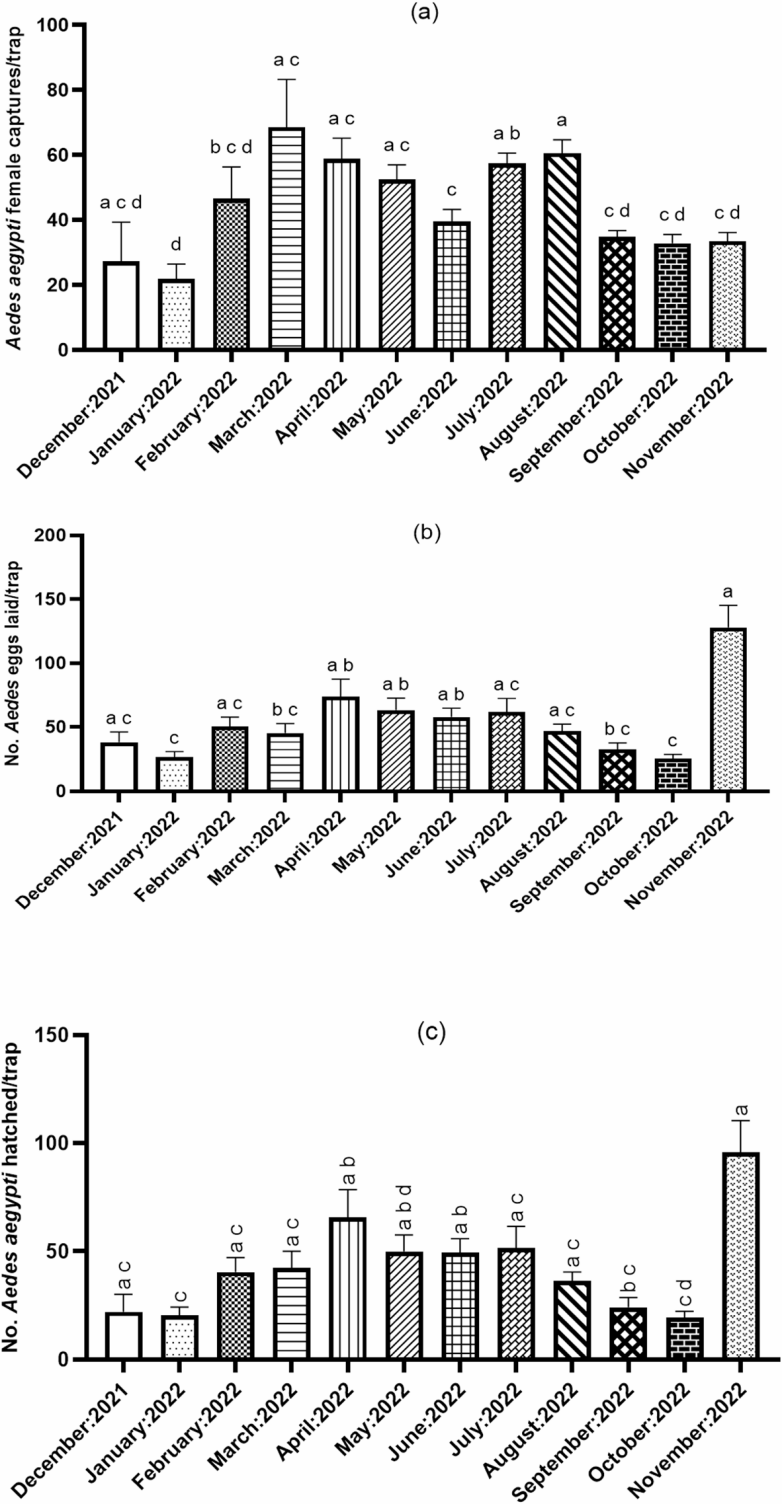
Densities of *Aedes aegypti* in Ukunda, coastal Kenya (Dec 2021–November 2022) (**a**) adult female, (**b**) number of *Aedes* eggs laid and (**c**) number of hatched adults.

A total of 52,121 adult mosquitoes were collected throughout the study period via CO_2_-baited BG-Sentinel traps (526 replicate traps). The captures predominantly comprised *A. aegypti* (35,109/52,121; 67.4%) followed by *Culex* spp. (16,970/52,121; 32.6%). Only two *A. simpsoni* s.l. and 40 *Eretmapodites chrysogaster* specimens were additionally recorded. Of the *A. aegypti,* 70% (24,713/35,109) were females. Abundance of *A. aegypti* males and females was highly correlated (Pearson correlation coefficient = 77.7%; t = 28.259, df = 524, p < 0.0001). Thus, further analysis of abundance trends and association with climate variables focused on females only (and applicable to males) and their role in pathogen transmission. There was a significant monthly variation in *A. aegypti* female abundance (χ^2^_13,514_ = 570.17;* p* < 0.0001; Fig. 2a). Additionally, female abundance exhibited distinct bimodal peaks in the months of March 2022 and August 2022 (Fig. 2a).

The ovitrap sampling yielded 10,257 *Aedes* eggs from 200 replicate traps (mean: 51.3 eggs/ovitrap) with an ovitrap positivity rate of 97.5% (195/200). Of these, 80.8% successfully hatched and were reared as adults, which were exclusively identified as *A. aegypti*. This translated to a strong positive correlation between no. of *Aedes* eggs and number of *A. aegypti* reared as adults (Pearson correlation coefficient 96.4%, t = 51.05, df = 198, *p* < 2.2e−16). Bimodal peaks in number of *Aedes* eggs occurred in April 2022 and November 2022, which, unexpectedly, was different from adult population peaks in March 2022 and August 2022. Similar to adult host-seeking captures, the number of *Aedes* eggs laid varied monthly* (*χ^2^_11,188_ = 226.86*;* p < 0.0001; Fig. 2b) just as the number of *A. aegypti* hatched as adults (Fig. 2c).

### Analysis of Blood meal source in field-collected fed females *A. aegypti*

**Fig. 3:**
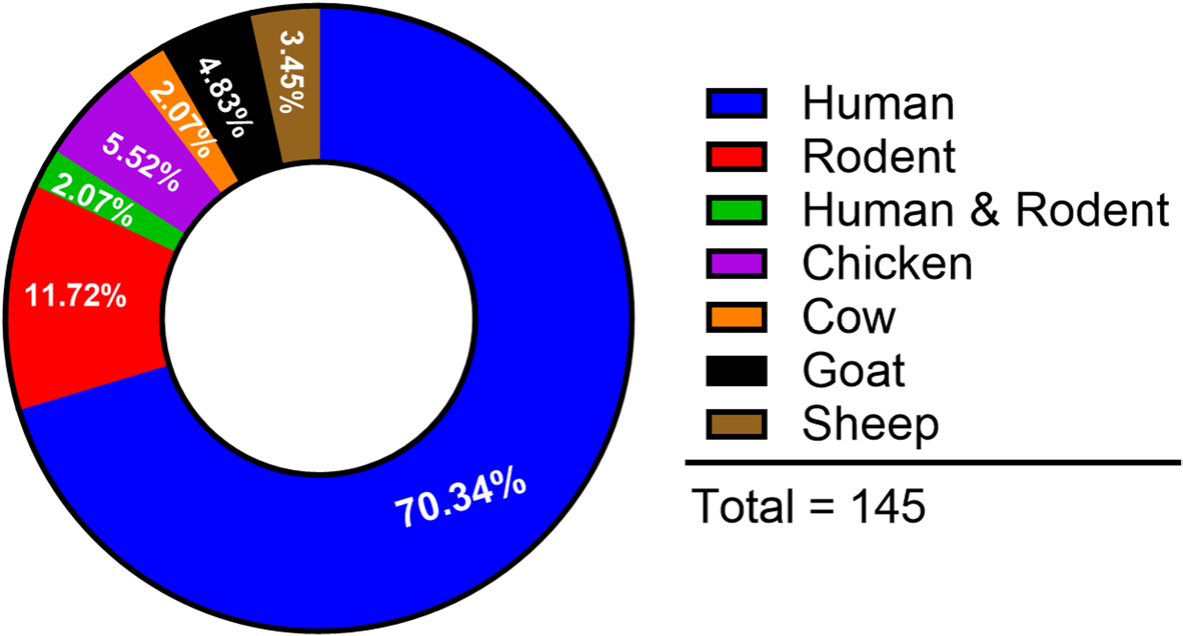
Trophic association of *Aedes aegypti* in urban Ukunda, coastal Kenya.

Overall, 165 blood-fed specimens were encountered comprising 0.67% of the total female captured in BG traps. Of these, 145 were analysed to identify host meal sources corresponding to 87.9% success rate. Human blood meals were detected in most samples with a human blood index of 0.72. Rodent blood meals were the next most abundant at a frequency of 13.8%; Low proportion of meals were obtained from livestock (15.9%) including chicken, goat, sheep and cattle (Fig. 3).

### Analysis of virus infection in field-collected *A. aegypti*

**Fig. 4:**
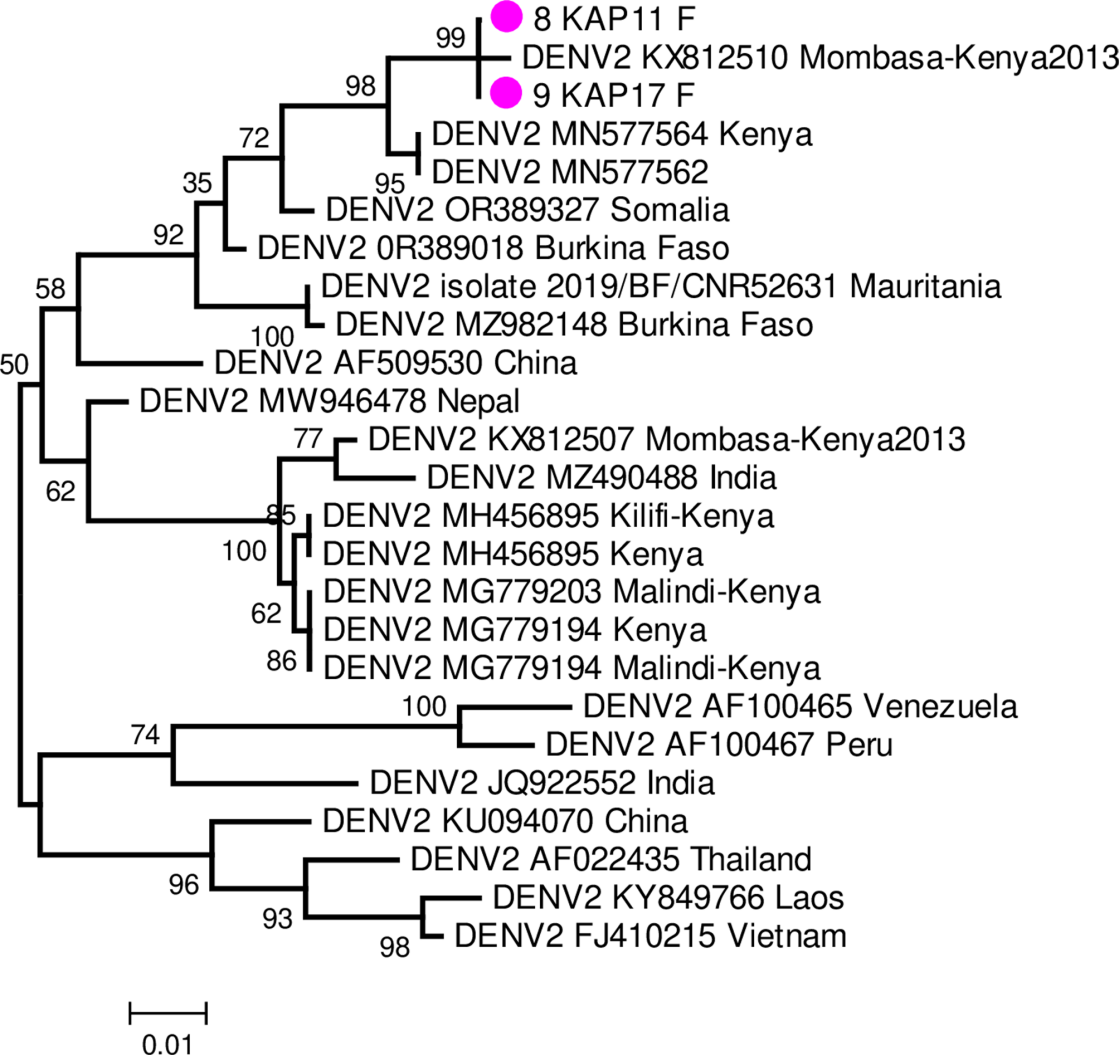
Maximum likelihood phylogenetic tree showing evolutionary relationship of dengue-2 viruses detected in this study based on 476–512 nt of the polyprotein gene sequences using a JC model, to selected ones in GenBank. Samples detected in this study are indicated with pink block circles. Scale bar indicates nucleotide substitutions per site. Sequences of the dengue-2 virus detected have been deposited in GenBank (accession numbers: PV404782-83).

A total of 6029 unfed females randomly selected from BG traps monthly collections were analysed for virus infection. None of the 256 pools analysed in cell culture and RT-PCR, tested positive for any of the classical viruses in the virus families tested. However, two blood-fed specimens (both had human DNA) individually tested, gave a positive signal for flavivirus primers. Both samples were encountered in May 2022 a period that did not coincide with peak abundance of female *A. aegypti*. Subsequent sequencing and bioinformatics query of GenBank revealed the detected viruses as both dengue serotype 2 virus. The sequenced fragment of the polyprotein gene 500nt was subjected to phylogenetic reconstruction with reference sequences. Phylogenetic analysis shows the isolates clustered strongly (bootstrap support = 98%) with dengue-2 virus isolated during the outbreak of 2013 in Mombasa and closely related to strains detected in febrile children in Ukunda (Fig. 4).

### Relationship between *A. aegypti* abundance and weather variables

**Fig. 5:**
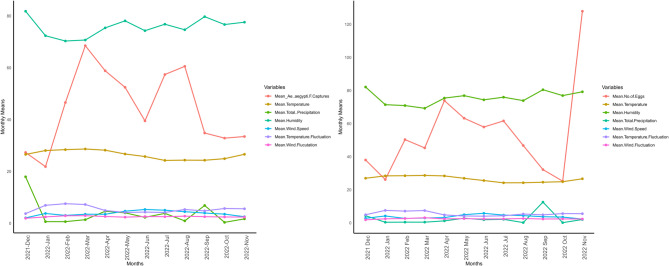
Time series of weather variables in relation to *A. aegypti* adult female densities (left panel) and *Aedes* eggs laid (right panel), in Ukunda (2021–2022), coastal Kenya.

The time series of the monthly distribution of *A. aegypti* female abundance against climatic variables are shown in Fig. 5. Throughout the study period, the range of the weather variables were temperature (23.7–29.3 °C), total precipitation (0–50.9 mm), relative humidity (66.4–85.5), wind speed (1.94–6.4 cm/s), DTR (1.9–10.0 °C) and DWR (1.4–4.4 cm/s). In general, there was no clear pattern observed between the climatic variables and female *A. aegypti* abundance. Neither temperature nor rainfall could be categorized into seasonal patterns as there was no discernible peak or sharp decline in the records analysed. However, temporal fluctuation in abundance of *A. aegypti* females exhibited sharp peaks in March and August 2022, in either case corresponding to an increase in temperature, temperature fluctuation, and a substantial drop in humidity levels (Fig. 5). In contrast, the number of *Aedes* eggs showed a peak in January 2022, then dropped sharply before rising again in November 2022 (Fig. 5). There was a positive relationship between mean wind, mean humidity and mean temperature with the number of eggs laid. However, total precipitation had a negative influence on the number of eggs laid, with high precipitation leading to decrease in egg densities per ovitrap (Fig. 5).

From the stepwise multiple linear regression model (Table S1) DWR and DTR were identified as the only variables explaining significant variation in the abundance of *A. aegypti* females. In the model, 59.9% (*p* = 0.02) of the variation in the abundance of *A. aegypti* females was explained by climatic variables (Table S1) attributed to DWR (82.9%) and DTR (17.1%). Random Forest model best explained the abundance trend in the number of eggs laid over the course of the study as per the performance metrics (MSE, mean squared error and R^2^, Table S2) explaining 82.3% variation in the data attributed to weather variables. Of these, the significant influencing factors positively were mean temperature (35.9%), mean wind speed (22.6%) and mean relative humidity (13.9%) and negatively total precipitation (27.6%).

## Discussion

We found *A. aegypti* as the predominant Stegomyia species during the study period using trapping methods that target both adults and eggs. *Aedes aegypti* displayed monthly variation in abundance with no discernible delineation by season and year-round presence indicating that this species is well adapted to thrive in the urban environments of Ukunda. Adaptation of *A. aegypti* in urban environments has been associated with a decrease in *Aedes* species richness^9,24,25^, consistent with our results. This variation together with high human feeding rates may reflect this mosquito species’ involvement in pathogen transmission^10,26,27^. The timing of peak adult abundance observed in BG traps did not coincide with thatof eggs laid in ovitraps. In part, the lack of overlap in peak adult and egg densities suggests that the availability of egg-laying sites may not have a major regulating effect on adult mosquito density^17^. Eggs laid may not represent a good proxy of *A. aegypti* adult population abundance. Instead, it may indicate different sensitivities they provide as risk indicators for dengue transmission since adult mosquito abundance is a better predictor for outbreaks than immature indices^28^. Our analysis provided additional evidence that climate variables could influence the behavioural attributes, including host-seeking and oviposition of adult females. Taken together, we demonstrate the value of longitudinal data describing mosquito population dynamics of benefit in predictive surveillance and risk assessment of arboviral diseases such as dengue.

Mosquito vectors are highly sensitive to meteorological variables among them temperature, precipitation, and relative humidity are known to affect several aspects of mosquito behaviour and ecology. Temperature but not rainfall had a significant effect on *A. aegypti* population dynamics^17^ but governed by specific threshold values^29^ with site- and species-specific differences^30^. A study in central Iran found that adult mosquito abundance correlated positively with temperature but negatively with relative humidity, rainfall and wind speed^31^. Recent studies showed that DTR could be more important in influencing dengue spread^32,33^, positively or negatively impacting virus susceptibility and survival in *A. aegypti* depending on the threshold. DTR can influence the frequency of blood meal imbibition in *A. aegypti*^5^, which is an endpoint of *A. aegypti* host-seeking behaviour. Our analysis provides additional evidence in support of DTR as an important predictor of adult female abundance dynamics. Furthermore, our data reveals the potential significant role of daily variation in wind speed (DWR), not considered previously in explaining the dynamics of *A. aegypti* and dengue transmission. Thus, our data suggests that short-term fluctuations in temperature and wind speed are important predictors of *A. aegypti* abundance. When analysed for effect of weather variables, the number of eggs laid had a positive relationship with mean wind, relative humidity and temperature consistent with the literature^34,35^. A negative correlation between the total precipitation and the number of eggs as shown in our data could occur via an indirect effect on flight dispersal that females must engage in to actively seek egg-laying sites. Warm, humid and calm conditions generally favour mosquito flight for egg-laying^34^. Wind speed is an important modulating factor of mosquito egg-laying via flight and dispersal ability, increasing values inversely affects trap catches^34^. Both *A. aegypti* adult densities and eggs laid varied monthly in Ukunda suggesting suitable environmental conditions impacting mosquito breeding habitats; improper waste disposal and abundance of discarded containers in the area^9^ could sustain mosquito breeding activities.

We detected dengue-2 virus in field collected *A. aegypti*, an indication of active circulation of the virus and potential endemicity in the studied area. This finding is significant given that the virus has rarely been detected in host-seeking female mosquitoes between epidemic episodes in Kenya. Low mosquito viral loads could affect virus detection or isolation without an outbreak. The detection of denv-2 serotype agrees with its circulation in human population as the dominant serotype at the time of study^3^. Virus detection in vectors could be confounded by monitoring strategy and our longitudinal design could have maximised detection between epidemics as recently reported in Benin^36^ and neighbouring coastal city of Dar es Salaam in Tanzania^37^. Two aspects emerge from our detection of the virus only in engorged mosquitoes that each had had a human meal. If human is the likely source of the virus, then the data provides entomological support for the notion that disease vectors may selectively feed on humans infected with virus pathogen consistent with results from the malaria parasite-*Anopheles* system^38^, however, this needs further evaluation. Blood represents a sensitive target to detect RNA viruses and in mosquito blood meals, it can be detected for up to 24 h after initial ingestion^39^. Blood-fed cohorts could be a sensitive target for implementing a low-cost molecular surveillance system to monitor circulation of the virus. Infection was detected during periods that did not coincide with peak abundance of vectors. Although the infection rates observed in this study were relatively low, the high density and year-round presence suggest potential significant risk of dengue transmission in the study area.

Low captures of blood-fed *A. aegypti* were recorded a finding not unexpected given that the use of CO_2_ baited BG traps mainly targets host seeking mosquitoes^19^. Even aspiration in potential resting sites including vegetation did not improve capture rates of blood-fed cohorts^8^, suggesting difficulty in trapping engorged *A. aegypti* in adequate numbers in some settings. Our analysis showed *A. aegypti* fed highly on humans (HBI > 70%) although we did not examine for monthly changes. The values are consistent with high human feeding rates reported for this species (reviewed in^26^). Previous studies in Kenya in other ecological settings found a lower human blood feeding index (< 0.5)^10,12,40^, the differences might be linked to the degree of urbanisation. Urbanisation is normally characterized by changes in socio-economic and cultural factors among others including a high human population density. It is not unexpected that *A. aegypti* human feeding increases in urban areas like Ukunda than rural or peri-urban environments with high human-vector contact. More feeding on humans likely reflects increased density relative to other vertebrates, a hallmark of urbanisation, and a recognised risk factor for occurrence of dengue epidemics. Non-human meals were readily observed on rodents and livestock hosts. This trend could expose humans to zoonotic pathogens associated with these hosts. While the factors that underlie this seeming opportunistic trend is unclear, a detailed study of the seasonal dynamics in host utilisation could be informative in risk assessment; diversion to non-humans could occur at specific times influenced by weather and accessibility to humans. Examining the relative importance of host availability to abundance in the observed feeding trend merits further elucidation.

The period of peak abundance of adult host-seeking females differed from that of *Aedes* eggs laid. The result suggests that these variables may provide different sensitivities as indicators of dengue transmission risk. In the absence of human case data, we were unable to explore the potential relationship between the estimated entomologic parameters and dengue disease occurrence. Estimates of egg laying has been suggested to be a better indicator of vector presence and pinpointing hot spots of infestation rather than disease, of value for targeted vector control activities^41^. On the other hand, variations in adult female densities offered a better entomologic indicator of dengue disease^41,42^. These hypotheses remain to be tested in our local settings. Exploring the connection between climatic factors, vectors and disease for early detection of outbreaks could aid planning of dengue surveillance, response and control activities. Extending our approach to estimate risk of the disease would be important given that establishing epidemiologically significant levels of entomological indices for dengue has been elusive^17^.

This study has several limitations. We found only a minor representation of *A. simpsoni* s.l. and other *Aedes* species in contrast to previous studies in Kenya^8,40^. BG trap as a monitoring tool for Stegomyia mosquitoes may not target species with the same efficiency. If these species are poorly attracted to BG traps, then they are likely to be detected through larval surveys in containers which is a limitation of the present study. Ovitrap collections exclusively detected *A. aegypti;* a finding which supports its abundance in this ecological setting. We relied on satellite-driven weather data which may differ from values obtained from local weather stations. However, satellite data are a good proxy for local data^43,44^ and we aimed to explore relationships with abundance and egg-laying patterns and assumed sufficient to demonstrate such relationships. Although meteorological variables are conducive to vector distribution, we do not claim causality on their effect on the abundance dynamic. The study was conducted in only Ukunda, which may limit the generalizability of the findings to other areas within the region, although we selected this urban site on account of previous evidence of dengue activity^3^. It is clear from our analysis that climatic variations alone do not explain the *A. aegypti* distributions, highlighting the role of other factors not considered in the present study. Other authors have highlighted the importance of mosquito age indicative of longevity as a sensitive indicator of risk^10,45,46^. These biological attributes may interact with a multitude of weather variables to affect *A. aegypti* dynamics and disease^2^ as well as human factors (e.g. herd immunity). Exploring this could benefit from longer-term data in sentinel sites spanning years in different ecological settings.

To conclude, we performed a longitudinal weekly monitoring of *A. aegypti* adult abundance and egg laying and showed monthly variation but year-round presence and no discernible effect of season in urban Ukunda. Female abundance correlated strongly with those of males with weather variables significantly explaining variation in abundance of female *A. aegypti* with DWR and DTR as the most influential factors. Egg laying was explained by weather variables and positively influenced by mean daily temperature, wind speed, and relative humidity but negatively by total precipitation. We demonstrate high human feeding tendencies of *A. aegypti* in this urban setting and detected dengue-2 virus RNA in *A. aegypti* indicating active circulation locally during the interepidemic period. Despite the low virus infection rates, the high density, human feeding rates and year-round presence indicate significant risk of dengue transmission in the study area. Together, we demonstrate the value of longitudinal data describing mosquito population dynamics of benefit in predictive surveillance of arboviral diseases such as dengue.

## Electronic supplementary material

Below is the link to the electronic supplementary material.

Supplementary Material 1

## Data Availability

Data is provided within the manuscript or supplementary information files. Sequences of the dengue-2 virus detected have been deposited in GenBank (accession numbers: PV404782-83).
