# Supplementary material for: Longitudinal surveillance of Aedes aegypti (Diptera: Culicidae) in urban coastal Kenya: population dynamics, blood feeding frequency and dengue virus infection rates
Source: Sci Rep. 2025 Jul 1;15:21787. doi: 10.1038/s41598-025-05408-z (PMC12216250; doi:10.1038/s41598-025-05408-z)
Supplement: Supplementary file 1 — Supplementary Material 1 [file 41598_2025_5408_MOESM1_ESM.docx]

**Table S1. Multiple Linear Regression Model explaining influential weather variables on abundance of adult female *Aedes aegypti***

| Term | Estimate | Std. Error | t value | P-value |
| --- | --- | --- | --- | --- |
| Intercept | -120.44 | 52.08 | -2.313 | 0.04604 |
| log_mean_DWR | 195.86 | 54.02 | 3.625 | 0.00552 |
| log_mean_DTR | -44.95 | 21.38 | -2.102 | 0.06487 |
| Model overall p-value: 0.01697 | | | | |
| Multiple R-squared: 0.5958 | | | | |

**Table S2. Model comparisons explaining influential weather variables on *Aedes* egg laying**

| **Model** | **MSE (Mean Squared Error)** | **R^2^(r squared)** |
| --- | --- | --- |
| **Random Forest** (Using average temp & average wind) | 637.37 | 82.3% |
| **Random Forest** (Using DTR &DWR) | 600.81 | 80.0% |
| **Multiple Linear Regression** (Using average temp & average wind) | 148.68 | 74.8% |
| **Multiple Linear Regression** (Using DTR &DWR) | 296.1 | 49.9% |
| **Lasso Regression** (Using average temp & average wind) | 148.99 | 74.8% |
| **Lasso Regression** (Using DTR &DWR) | 148.99 | 49.8% |
| **Ridge Regression** (Using average temp & average wind) | 201.02 | 66.0% |
| **Ridge Regression** (Using DTR &DWR) | 201.02 | 45.8% |

DWR, daily range in wind speed; DTR, daily temperature range
